# Combined Orthodontic and Surgical Approach in the Correction of a Class III Skeletal Malocclusion with Mandibular Prognathism and Vertical Maxillary Excess Using Bimaxillary Osteotomy

**DOI:** 10.1155/2013/797846

**Published:** 2013-12-22

**Authors:** George Jose Cherackal, Eapen Thomas, Akhilesh Prathap

**Affiliations:** ^1^Department of Orthodontics, Pushpagiri College of Dental Sciences, Medicity, Tiruvalla, Kerala 689 107, India; ^2^Department of Oral and Maxillofacial Surgery, Pushpagiri College of Dental Sciences, Medicity, Tiruvalla, Kerala 689 107, India

## Abstract

For patients whose orthodontic problems are so severe that neither growth modification nor camouflage offers a solution, surgery to realign the jaws or reposition dentoalveolar segments is the only possible treatment. Surgery is not a substitute for orthodontics in these patients. Instead, it must be properly coordinated with orthodontics and other dental treatments to achieve good overall results. Dramatic progress in recent years has made it possible for combined surgical orthodontic treatment to be carried out successfully for patients with a severe dentofacial problem of any type. This case report provides an overview of the current treatment methodology in managing a combination of asymmetrical mandibular prognathism and vertical maxillary excess.

## 1. Introduction

The correction of dentoskeletal malocclusions has always had a threefold goal of achieving functional efficiency, structural balance, and aesthetics [1, 2]. The physical health of patients with severe malocclusion may be altered or compromised in various ways such as inducing masticatory dysfunction, speech disorders, upper airway resistance, compromised oral hygiene, and temporomandibular joint dysfunction. Nevertheless, above all in a modern society, the aesthetic aspect of severe malocclusion with its related psychosocial impact is more important than the associated physical problems [3]. The positive effects of having an attractive face on an individual's mindset are clear, in terms of self-confidence and self-respect.

In cases of severe malocclusion with dentoskeletal discrepancy there are generally only three possible therapeutic options: early modification of growth, orthodontic camouflage through dental compensation, or combined orthodontic and surgical (orthognathic) repositioning of the jaw bases [1]. In recent years, an increasing number of patients elect to undergo orthognathic treatment to correct severe malocclusion that is not susceptible to a comprehensive orthodontic solution. This case report presents the treatment of an adult girl with skeletal discrepancies in all three planes of space: sagittal (Class III malocclusion), vertical (vertical maxillary excess) and transverse (facial asymmetry).

## 2. Case Report

**Figure 1:**
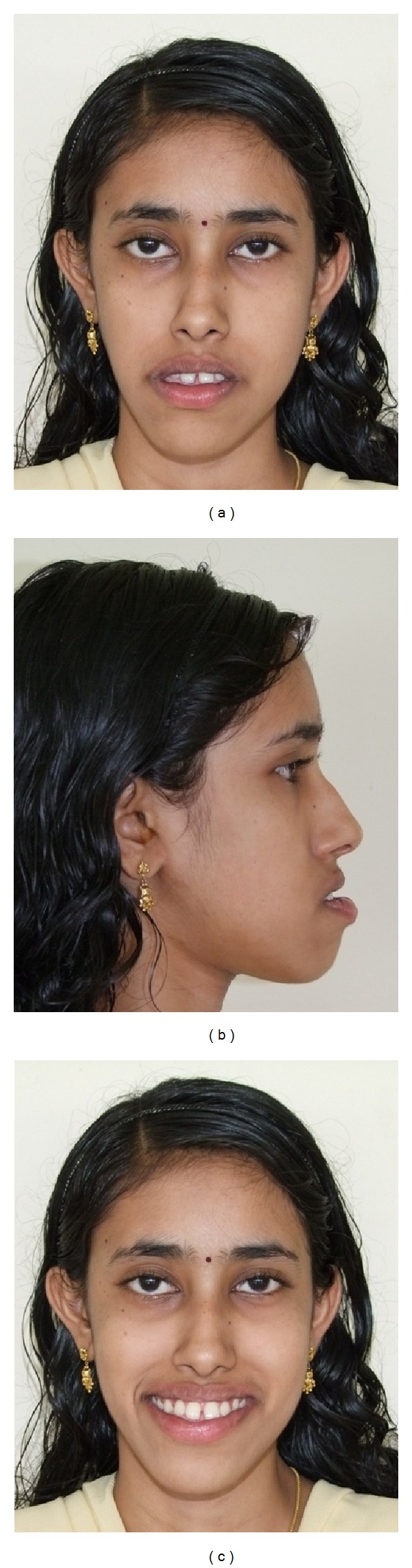
Pretreatment face: (a) frontal, (b) profile, and (c) smile.

**Figure 2:**
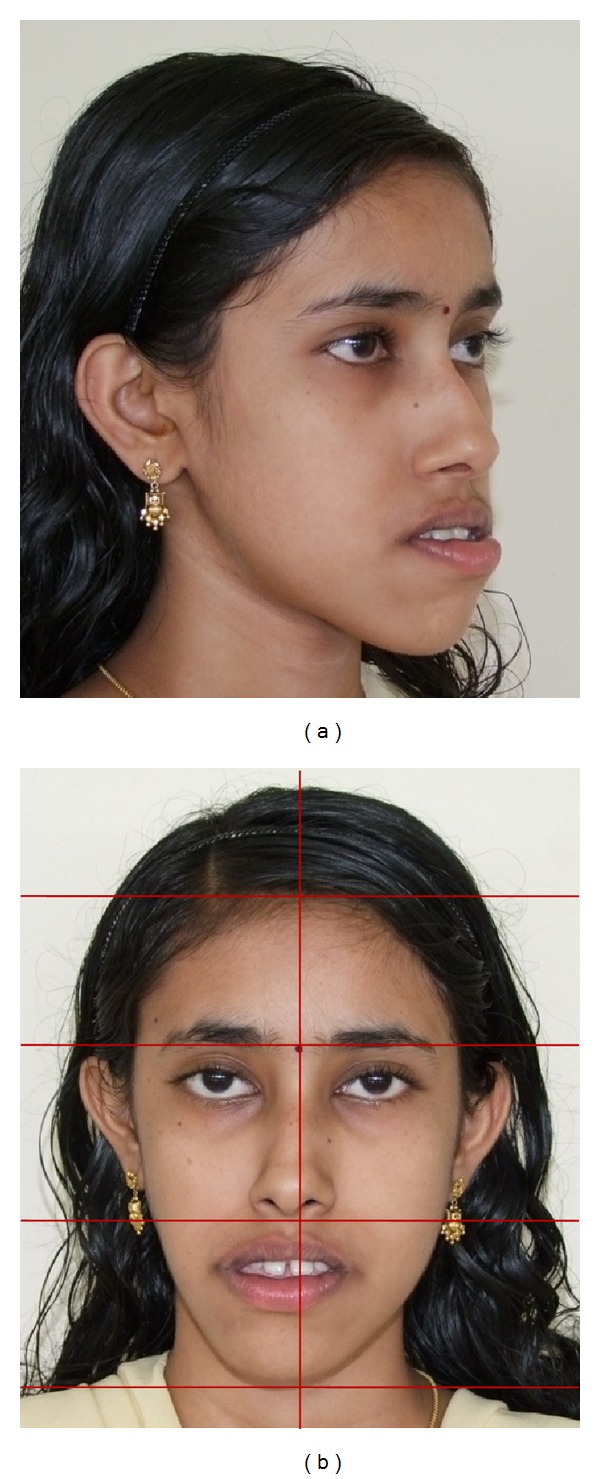
Pretreatment face: (a) oblique, and (b) vertical proportions and symmetry.

**Figure 3:**
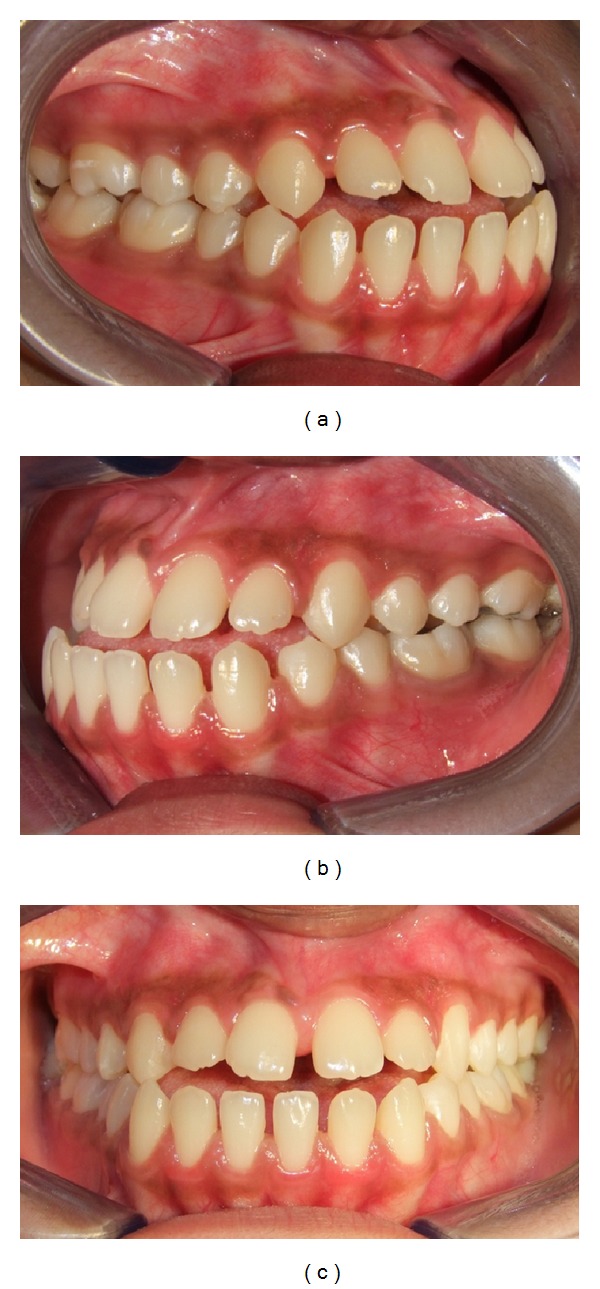
Pretreatment occlusion: (a) right, (b) left, and (c) front.

**Figure 4:**
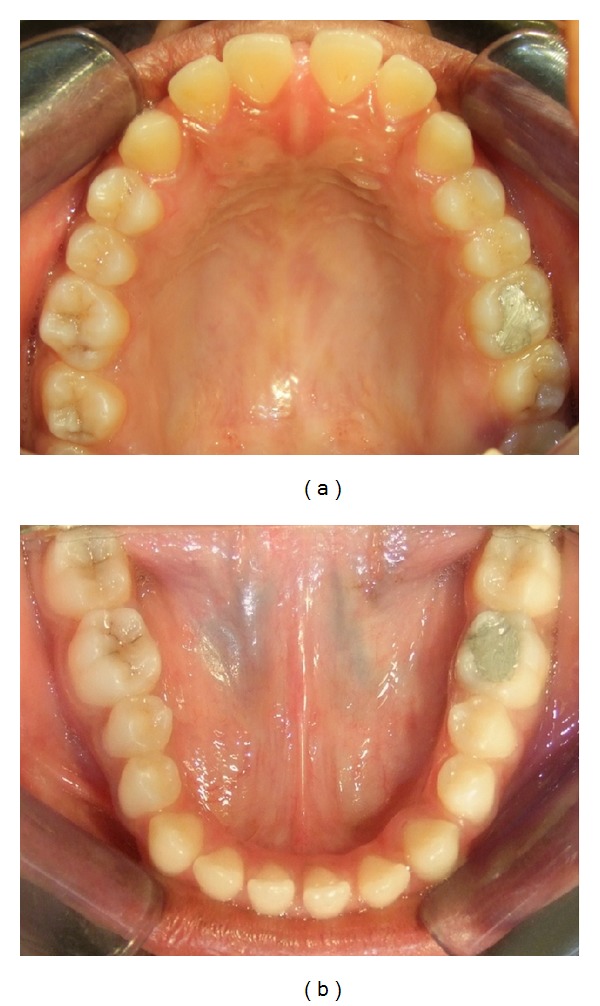
Pretreatment: (a) upper, and (b) lower arch.

An adult female patient with a chronological age of 19 years and 4 months and an ectomorphic body type reported to the Department of Orthodontics, Pushpagiri College of Dental Sciences, with the chief concern of unattractive facial appearance due to forwardly placed lower jaw and teeth. (Figures 1–4). Her parents pointed out that she was greatly dissatisfied by her looks. There was no relevant familial history pertaining to skeletal Class III malocclusion, nor any pertinent medical history.

### 2.1. Clinical Characteristics

Extraoral clinical examination in frontal view exhibits the patient's face to be leptoprosopic (oval); paranasal areas were deficient; lips were potentially incompetent with excessive lower lip vermilion exposure, and facial asymmetry was evident with the lower border of mandible moderately shifted to the right. Her growth pattern was predominantly vertical and there was increased incisor and gingival visibility at smile. Examination of vertical facial proportions (Figure 2) revealed that both her midfacial and lower anterior facial heights were increased which corroborates an increased display of teeth and gingiva. Lateral view and oblique view showed pronounced mandibular prognathism, with a concave profile, an acute nasolabial angle, an everted and hypotonic lower lip, reduced labiomental fold, a long chin-throat length with a well-defined inferior border of the mandible, and an acute lip-chin-throat angle.

Intraorally, the molar relationship was Class III with a complete anterior crossbite, along with a reverse overjet of 2 mm (Figure 3). There was generalized interdental spacing amongst her anterior teeth and the incisors were flared, combined with an anterior open bite. These characteristics may point to a large tongue. A lower dental midline discrepancy was present due to mandibular shift to the right. She had recently undergone endodontic treatment for her lower left first molar.

**Figure 5:**
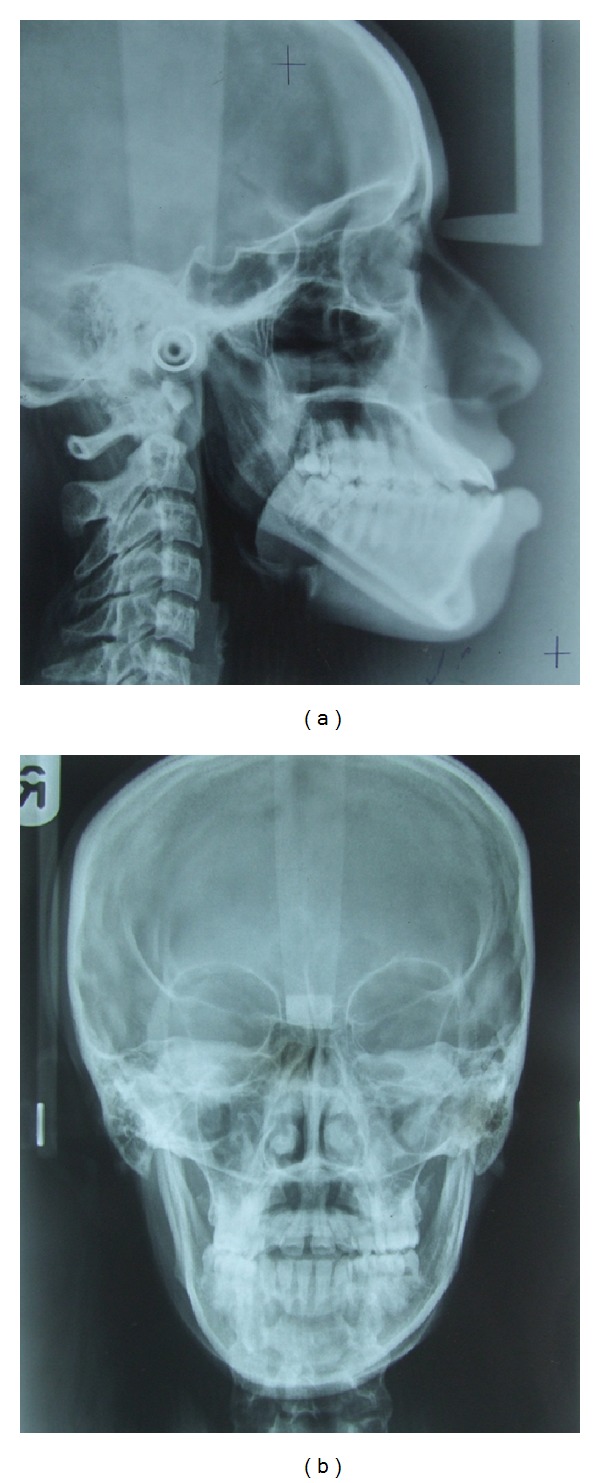
Pretreatment: (a) lateral, and (b) PA cephalograms.

**Figure 6:**
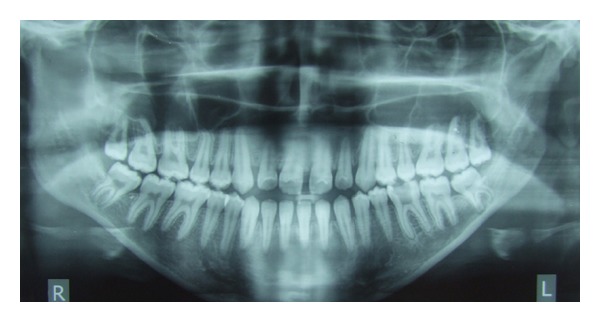
Pretreatment orthopantomogram (OPG).

Pretreatment radiographic records included lateral and posteroanterior (PA) cephalograms and orthopantomogram (OPG) (Figures 5-6). Cephalometric analysis (Table 1) revealed a Class III skeletal base with mandibular prognathism, increased maxillary skeletal and dental height, increased vertical chin height, and upper anterior proclination with compensatory lower anterior retroclination.

### 2.2. Treatment Plan

After a joint clinic discussion with the Department of Oral and Maxillofacial Surgery, Pushpagiri College of Dental Sciences, Le Fort I maxillary impaction in combination with mandibular setback by bilateral sagittal split osteotomy (BSSO), with presurgical and postsurgical orthodontics, was planned in order to achieve facial aesthetics and a functionally optimum occlusion. During BSSO an asymmetrical setback with more orientation to the left was planned in order to correct the shift of the lower jaw and midline. Vertical reduction genioplasty for correction of the chin was considered, subjected to the changes, at the time of surgery or as a secondary procedure. Lower lip augmentation to reduce the thickness and vermillion display along with rhinoplasty later was planned, if needed, after thorough posttreatment evaluation. Therapeutic extractions of all third molars were to be done prior to surgery.

### 2.3. Presurgical Orthodontics

**Figure 7:**
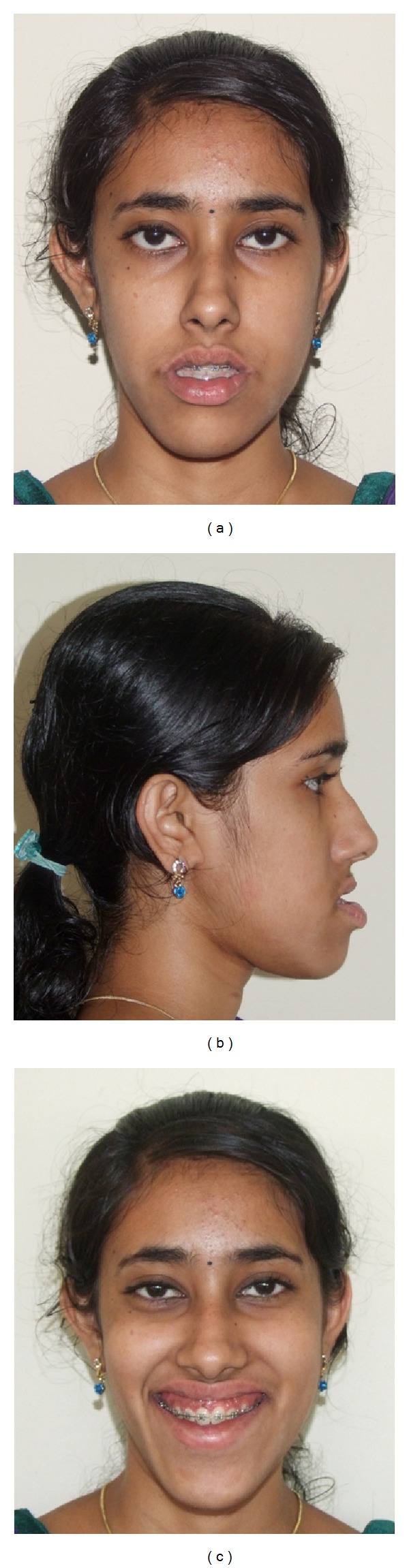
Presurgical face: (a) frontal, (b) profile, and (c) smile.

**Figure 8:**
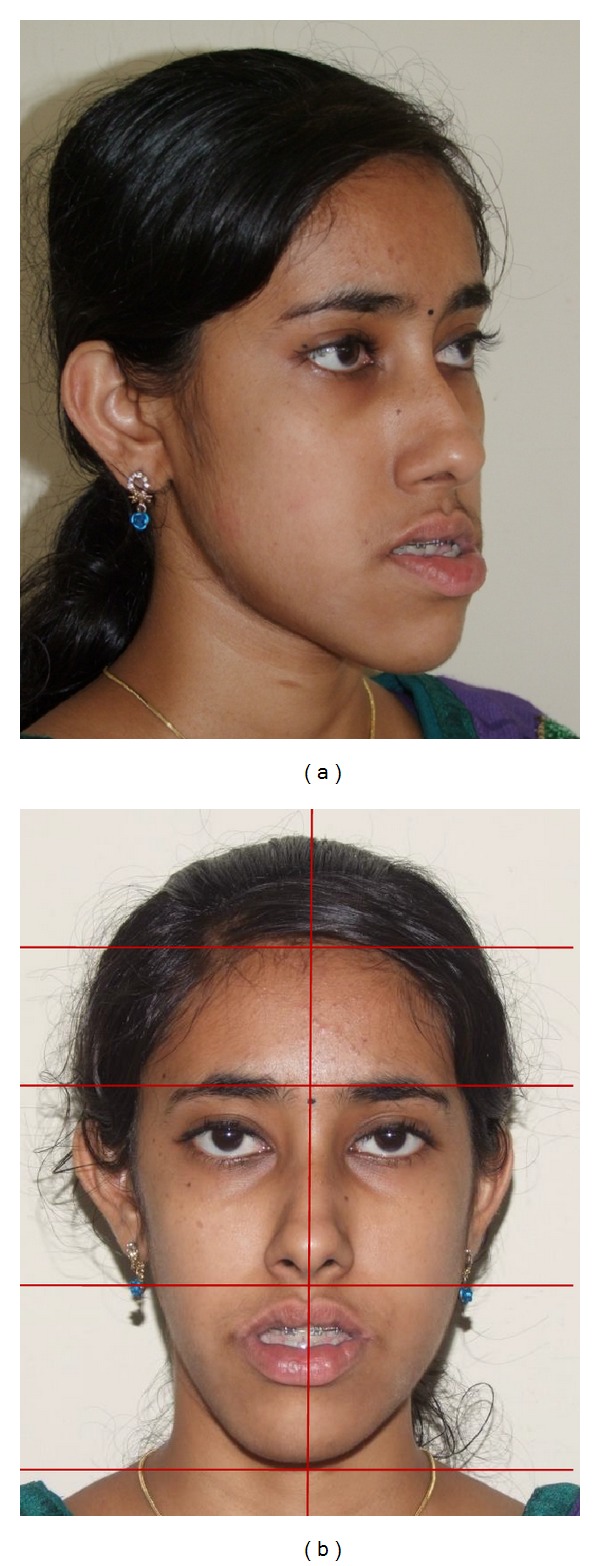
Presurgical face: (a) oblique, and (b) vertical proportions and symmetry.

**Figure 9:**
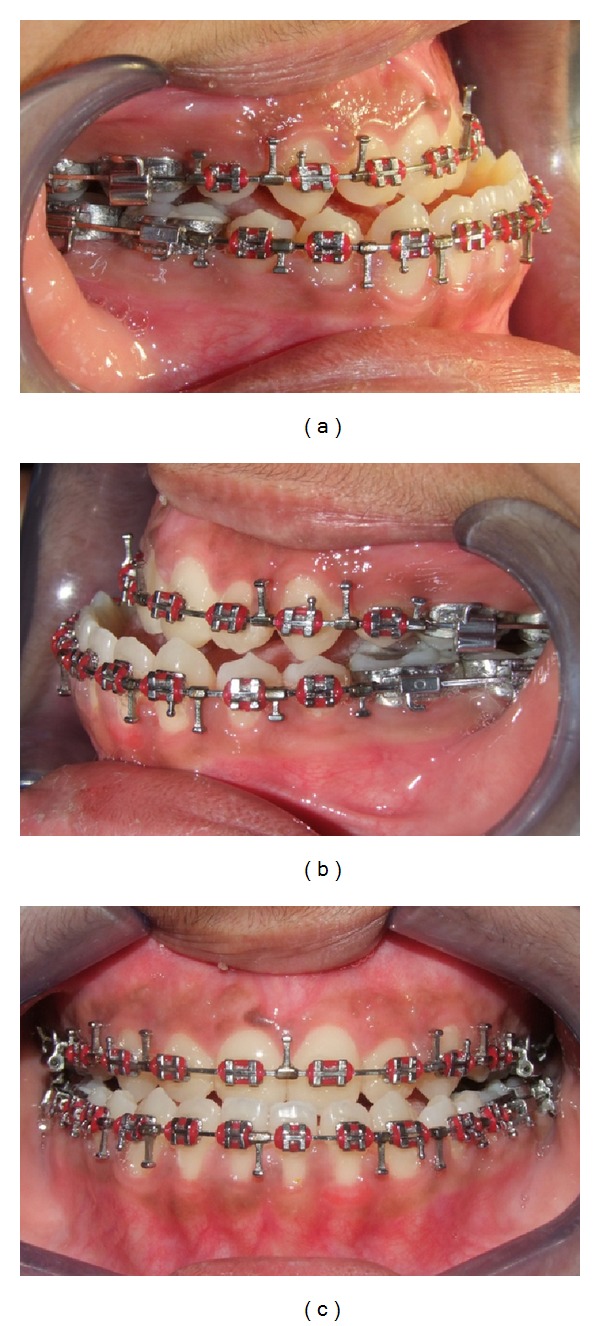
Presurgical occlusion, (a) right, (b) left, and (c) front.

**Figure 10:**
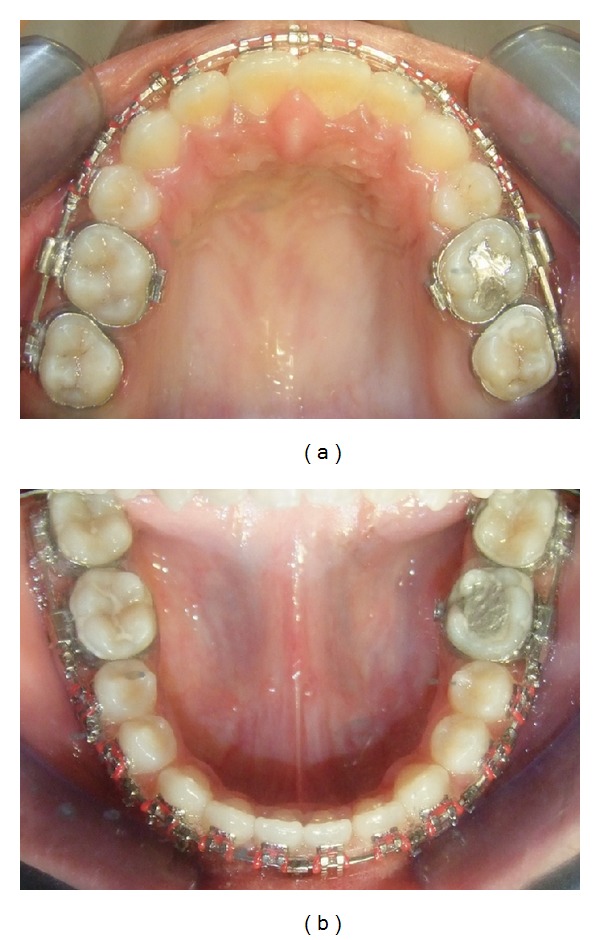
Presurgical: (a) upper, and (b) lower arch.

**Figure 11:**
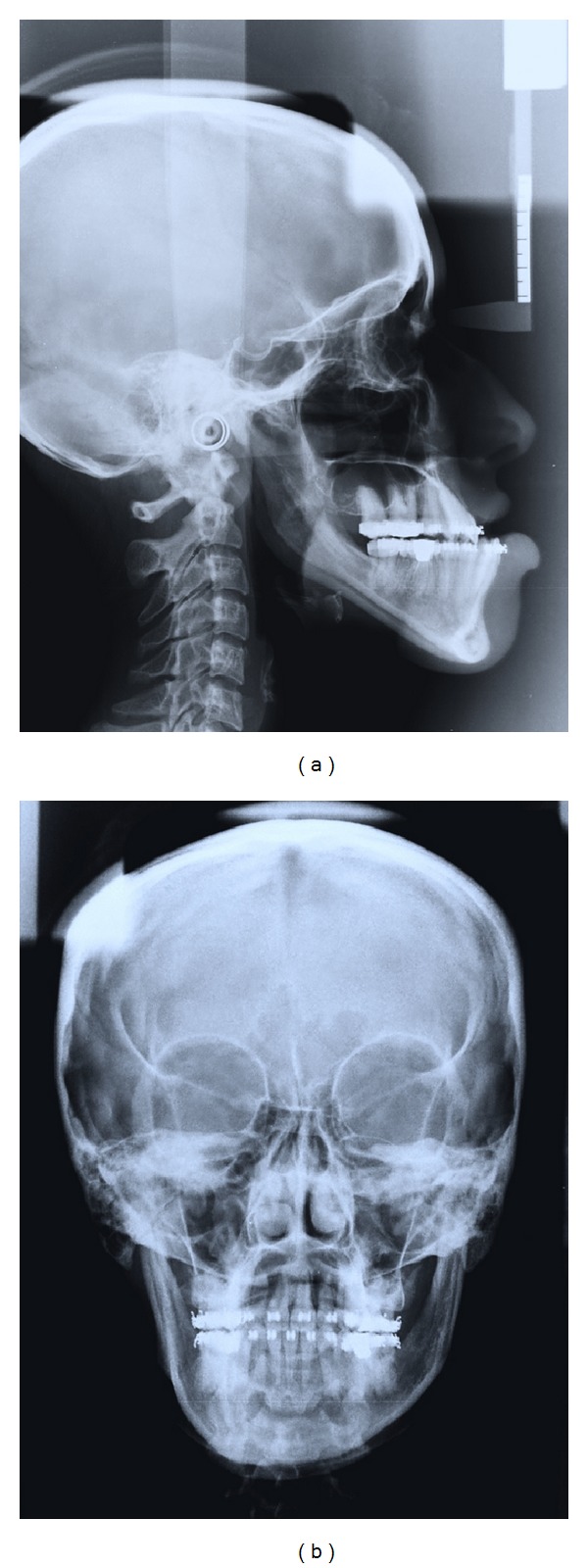
Presurgical: (a) lateral, and (b) PA cephalograms.

**Figure 12:**
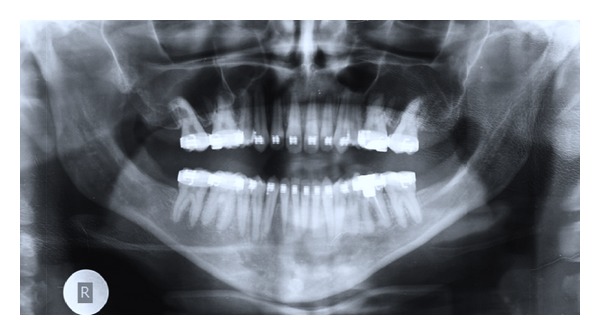
Presurgical orthopantomogram (OPG).

After initial restorative and prophylactic measures, presurgical orthodontics was begun with 0.022′′ × 0.028′′ Roth preadjusted edgewise prescription appliance. Intra-arch levelling and aligning were achieved, and spaces were consolidated in both the arches. To achieve sufficient decompensation and ideal maxillary incisor inclination therapeutic extractions of upper first premolars were done, which was followed by controlled retraction of maxillary anterior segment with loop mechanics. Maxillary and mandibular arches were aligned up to 0.021′′ × 0.025′′ stainless steel wire and arch compatibility was established between upper and lower arches (Figures 7, 8, 9, and 10). In due course upper and lower third molars were extracted to facilitate the orthognathic surgery. At the end of the presurgical phase radiographic records were repeated and compared (Figures 11 and 12).

**Figure 13:**
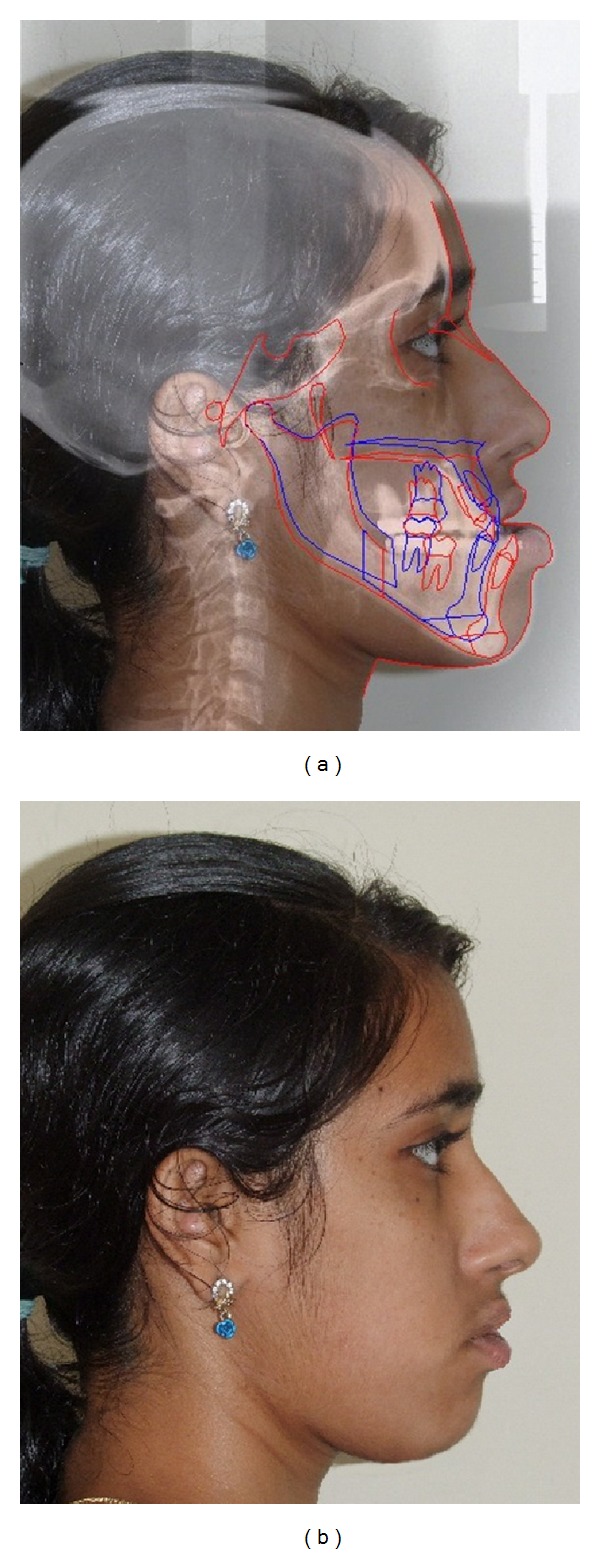
(a) Digital simulation of intended jaw movements (blue) and (b) predicted photo.

In order to assess the practical considerations and further predict the results of the planned surgical approach, cephalometric prediction tracing was done both manually using the template method and with computer image prediction. In template method, skeletal profiles of maxilla and mandible were traced on an acetate paper. Profile tracing was then duplicated and transferred to a thin cardboard. This outline was then cut to produce a cardboard template. From these templates, trial sections were made until desirable location and amount for osteotomy were found. The cut sections of both maxilla and mandible were then fitted back to the tracing in desired occlusal relation. Finally, soft tissue outline can be traced in regard to the reference ratios, and the probable postsurgical changes were checked [4]. Later, computer-based analysis was done wherein cephalometric landmarks were digitized and the surgical repositioning was monitorized. Measurements, calculations, and analyses were performed using Facad (Ilexis AB, Sweden). The obtained data was incorporated into prediction algorithms to provide single-line profile drawings predicting the final treatment goal (Figure 13). In the Maxilla, a 5 mm of superior repositioning along with an advancement of about 3 mm, was sufficient enough to reduce the gingival show and improve the para-nasal hollowing. In Mandible, 8 mm of setback brought about good posterior intercuspation with an aesthetically pleasing profile.

**Figure 14:**
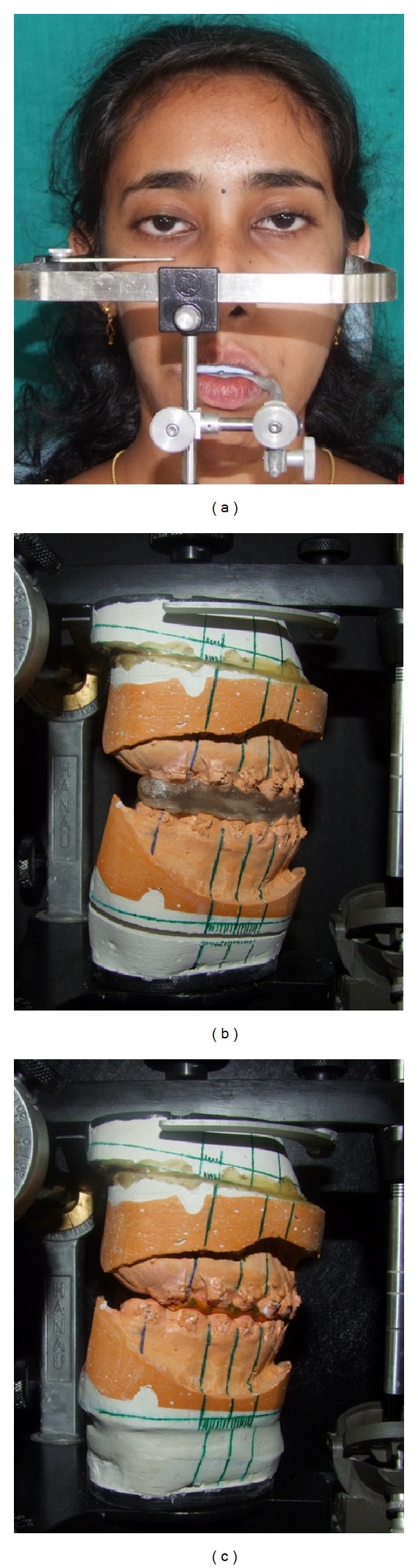
(a) facebow transfer and model surgery with (b) intermediate and (c) final occlusal splints.

Cast prediction or model surgery and fabrication of occlusal splints for use at surgery were the next steps in the planning sequence. Since both jaws were to be repositioned, the maxillary and mandibular dental casts were mounted on a semiadjustable articulator with the aid of facebow transfer (Figure 14(a)) and bite registration taken with the patient's jaws in the retruded contact position or centric relation. Model simulation of the anticipated surgical movement was performed next. The individual dental casts were repositioned, simulating the movements of the jaws as depicted by the manual and digital prediction. An intermediate acrylic occlusal splint was fabricated after the maxillary cast was repositioned on the articulator (Figure 14(b)). The mandibular cast was then repositioned to oppose the maxillary cast, simulating the final position of the jaws at surgery. Based on this position the final occlusal splint was then fabricated (Figure 14(c)).

### 2.4. Surgical Procedure

**Figure 15:**
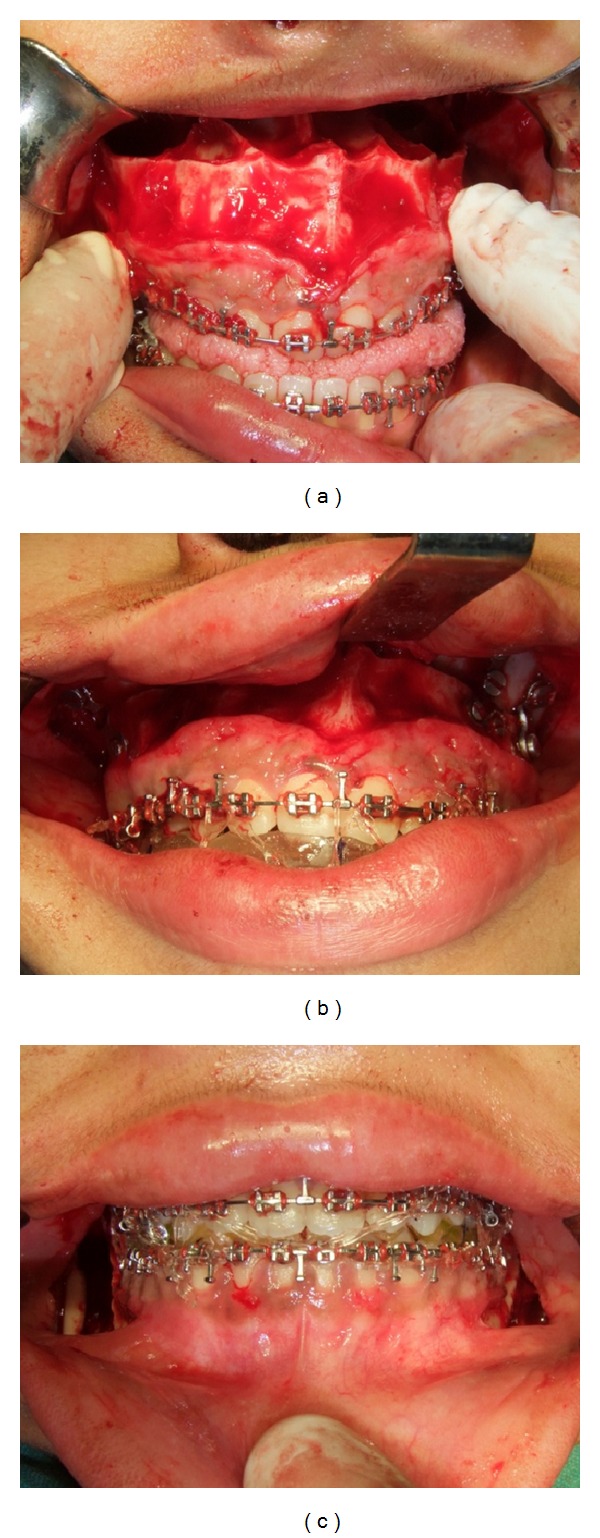
(a) Le Fort I maxillary osteotomy, (b) rigid fixation with bone plates and intermediate occlusal splint, and (c) BSSO with mandibular setback and final occlusal splint.

Le Forte I maxillary impaction was carried out initially as decided, with utilization of modified hypotension to decrease blood loss in anaesthesia [5]. The maxilla was repositioned 5 mm superiorly and 3 mm anteriorly (Figure 15(a)). Bilateral sagittal split osteotomy with short lingual split was carried out using surgical saws, and the mandible was set back by 8 mm (Figure 15(c)) after compensating for the mild autorotation due to maxillary impaction [6, 7]. The BSSO setback was performed asymmetrically with more orientation to the left so as to aid in the correction of mandibular shift, thereby improving facial and dental symmetry. Rigid type fixations were used in both jaws using four-hole miniplates and screw on both sides (Figure 15(b)). Genioplasty was to be performed as a secondary procedure, if needed, after thorough evaluation of postsurgical healing. Intermaxillary guiding elastics were engaged on the archwire hooks for 14 days during the immediate postoperative phase. The patient was followed up closely after the procedure and was guided to perform opening and lateral jaw movements.

### 2.5. Postsurgical Orthodontics

**Figure 16:**
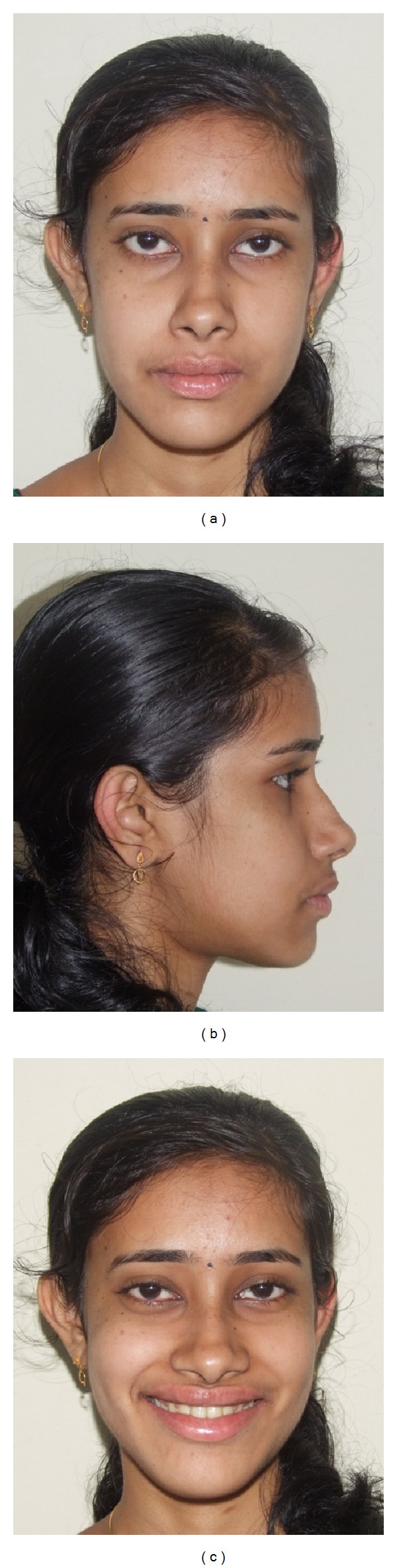
Posttreatment face: (a) frontal, (b) profile, and (c) smile.

**Figure 17:**
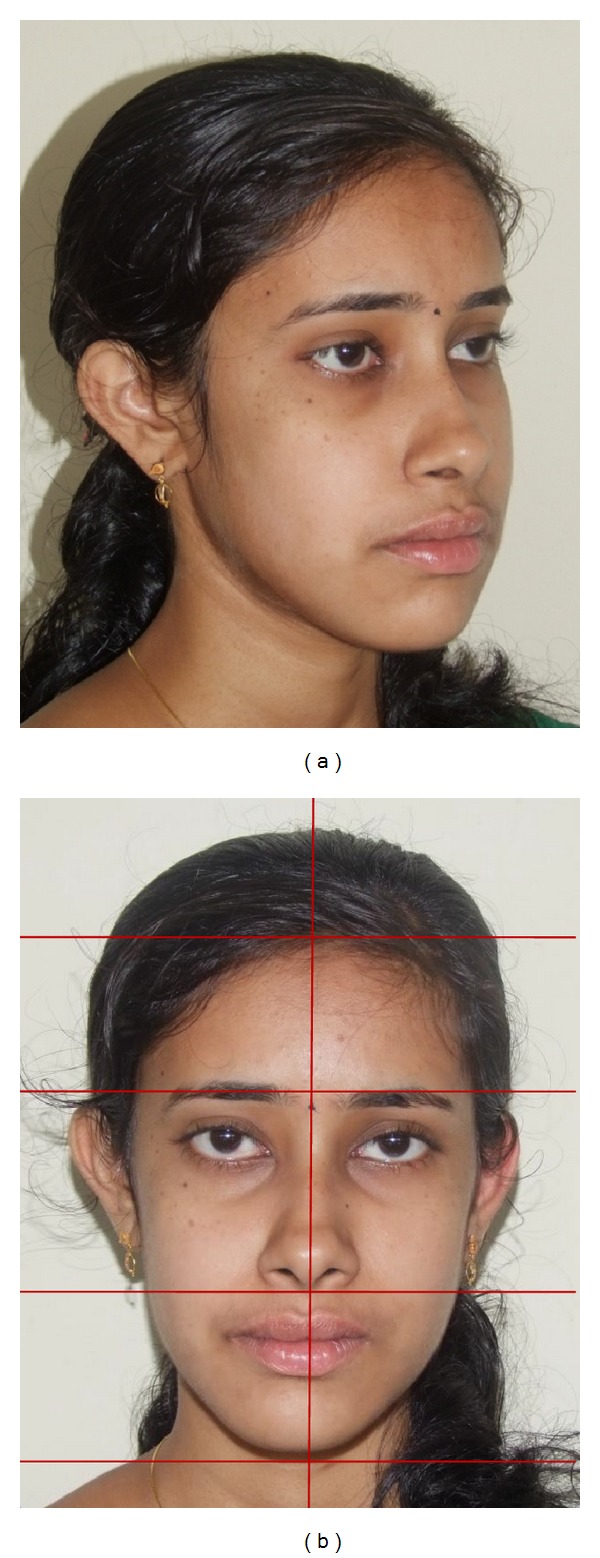
Posttreatment face: (a) oblique, and (b) vertical proportions and symmetry.

**Figure 18:**
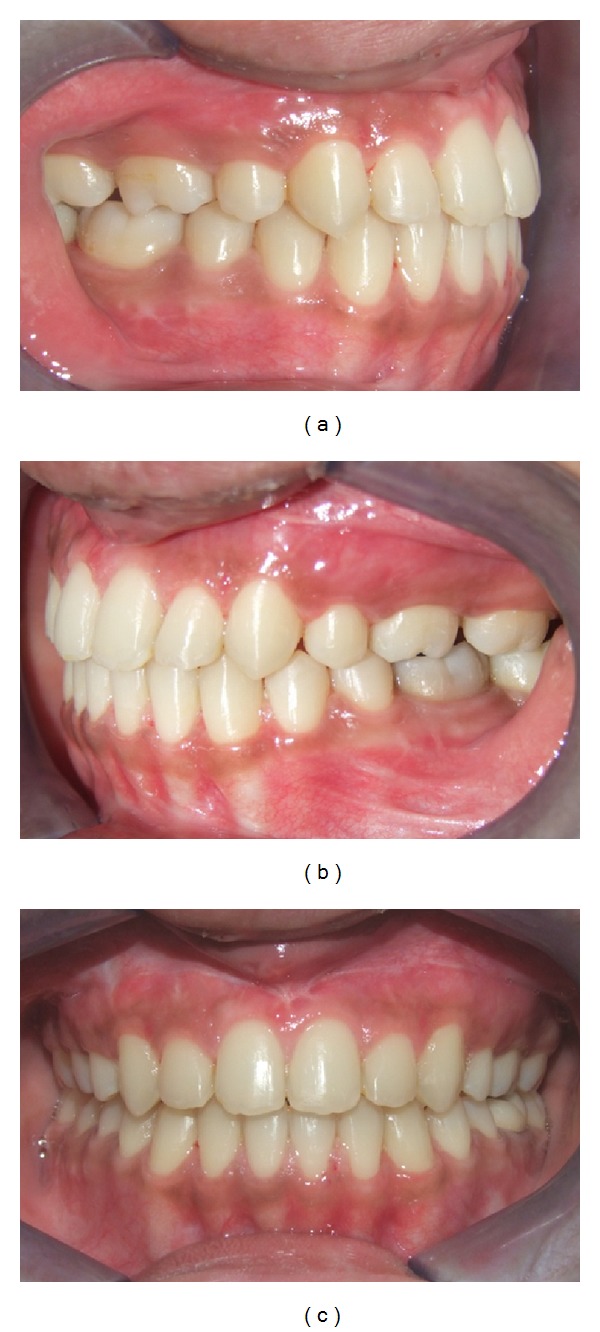
Posttreatment occlusion: (a) right, (b) left, and (c) front.

**Figure 19:**
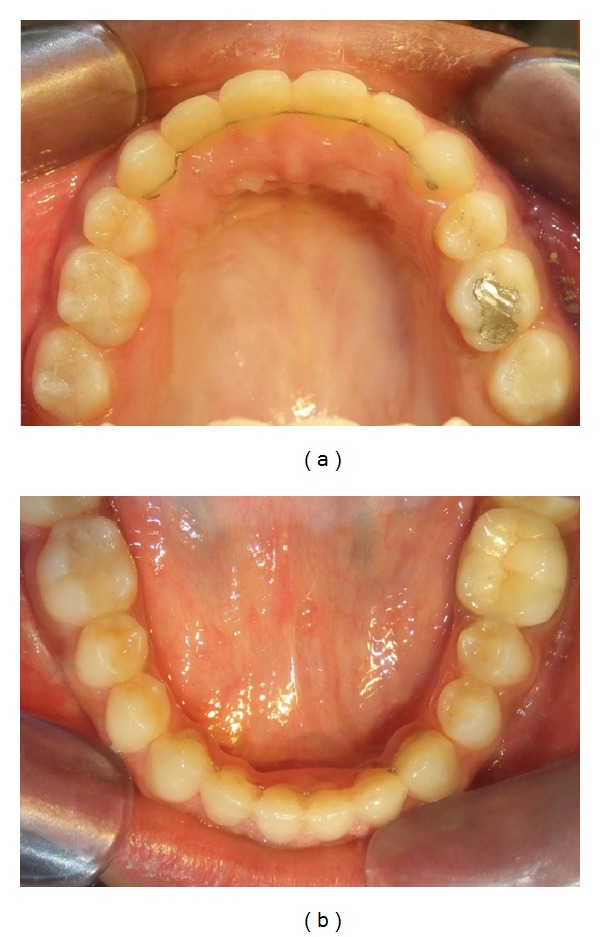
Posttreatment: (a) Upper, and (b) lower arch.

**Figure 20:**
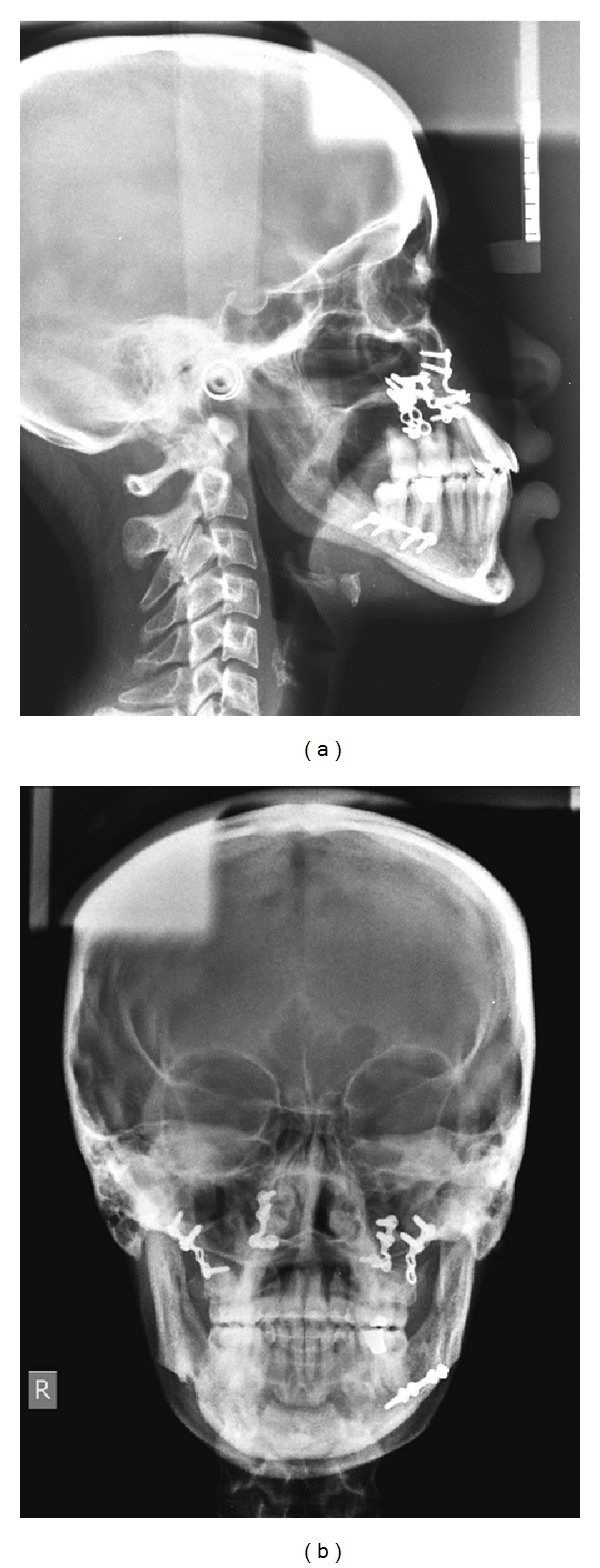
Posttreatment: (a) lateral, and (b) PA cephalograms.

**Figure 21:**
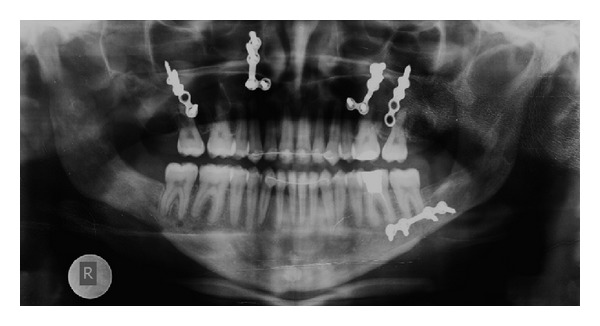
Posttreatment orthopantomogram (OPG).

**Figure 22:**
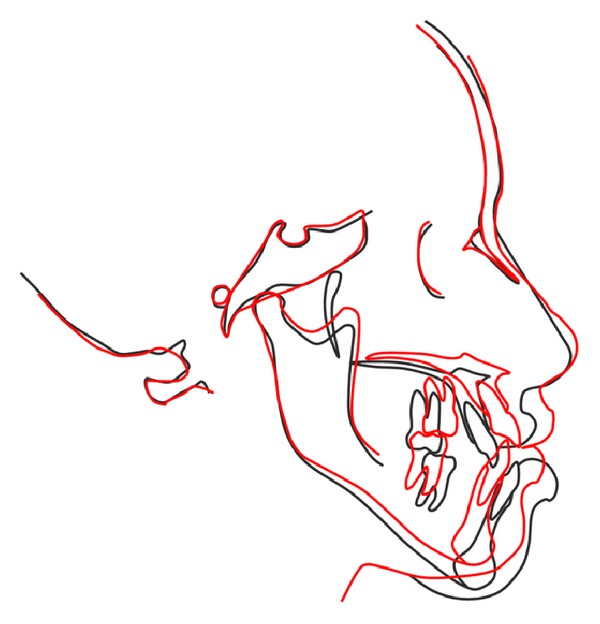
Cephalometric superimposition: before (black) and after (red).

Active orthodontic treatment was resumed 4 weeks after surgery when a satisfactory range of jaw movement was achieved and there was good bone healing and tolerance. The goal was to achieve ideal occlusal relationships, in terms of canine class, molar class, overjet, overbite, and coincidence of the dental midlines. During postsurgical orthodontics arch wires were sequentially changed from 0.017′′ × 0.025′′ NiTi to 0.019′′ × 0.025′′ SS wires. Closure of any residual diastema was achieved, and intercuspation was perfected by segmental settling with short inter-maxillary elastics. In the course of the postsurgical phase, the only eventful occurrence was with her mandibular right bone plate loosening and the upper left bone plate screw getting exposed into the vestibule, both of which were removed in the due course. After seven months of intervention, fixed appliances were debonded (Figures 16, 17, 18, and 19) and posttreatment retention phase was initiated with both fixed retainers and removable retention plates. Posttreatment radiographs were taken (Figures 20-21) and evaluated for treatment changes by superimposition (Figure 22). Pre- and posttreatment cephalometric values have been compared in Table 1. The overall treatment duration was 34 months.

## 3. Discussion

Establishing common objectives and expectations concerning the outcome of proposed surgical orthodontic therapy is a crucial part of the treatment planning process. Therefore a multidisciplinary team approach, in recommending two-jaw surgery to the patient, such as in this case, requires clinical judgment and experience. After thorough evaluation the presurgical phase of orthodontic treatment was initiated with the aim to achieve ideal inter- and intra-arch coordination, with each tooth in the correct position, always bearing in mind the goals of the subsequent surgical repositioning. In this case both manual and digital cephalometric predictions were employed at the end of presurgical phase. Predictions of changes in the frontal view still are artwork rather than science, but current computer prediction programs do a good job in predicting profile changes [8]. Though computer simulations are only as good as the algorithms on which they are based, these have considerably improved in recent years, allowing reliable adjustment of the hard-to-soft tissue ratios. However, one drawback could be that these ratios often do not take into account potential long-term skeletal relapse.

Bimaxillary osteotomy has a greater potential to decrease or increase anterior face height compared to one-jaw osteotomies, and the soft tissues may be affected by relaxation or stretching. In most patients, such as in this case, this is performed because of an excessive vertical dimension of the lower face [9]. These surgical movements have been shown to have excellent postsurgical stability when upward and forward movements of the maxilla are combined with lower border ramus osteotomies, thus preventing excessive forward rotation of the mandible [10, 11]. The relative amount of maxillary advancement and mandibular setback should also be planned according to the desired profile changes and should take into account the extent to which the soft tissues follow the hard tissue relapse in the long term. In this case since there was an evident paranasal hollowing, it was decided to advance the maxilla marginally to obtain facial fullness and as well limit the degree of mandibular setback within stable limits. During mandibular osteotomy a differential setback was performed which significantly improved the facial symmetry. Previous studies have shown that asymmetric setback of the mandible with intraoperative manual positioning of the condyle was favourable and does not significantly change the articular disc position in the condylar fossa [12, 13]. During the post surgical phase there was notable change in the soft tissue contour of the nose and lip. If necessary, both reduction cheiloplasty to address increased anterior projection, vertical lip height, and wet-vermilion show, and rhinoplasty for refinement of the tip and lateral nasal walls were to be done, along with genioplasty, as a secondary procedure. Cheiloplasty will address an increased anterior projection, vertical lip height and wet-vermilion show. Rhinoplasty can be done for refinement of the nose tip and lateral nasal walls, and genioplasty with lateral shift can address the chin asymmetry [14].

Postsurgical orthodontics was done in this case for 7 months, and it primarily involves finalization of the occlusion and retention. The duration of the final orthodontic phase depends on the degree of preparation achieved during presurgical treatment [15]. It is, however, important to stress that good dental retention contributes to maintaining the final occlusion that was achieved surgically, guaranteeing occlusal stability, which will surely have positive repercussions on the final hard tissue stability.

## Figures and Tables

**Table 1:** Cephalometrics for orthognathic surgery.

Variable	Clinical norm	Pretreatment	Posttreatment	Description
Horizontal (skeletal)				
N-A-Pg	2.6 ± 5.1 mm	−2°	7°	Convexity
N-A	−2.0 ± 3.7 mm	5 mm	9 mm	Maxillary position
N-B	−6.9 ± 4.3 mm	13 mm	5 mm	Mandibular position
N-Pg	−6.5 ± 5.1 mm	15 mm	10 mm	Position of chin

Vertical (skeletal and dental)				
N-ANS	50.0 ± 2.4 mm	56 mm	51 mm	Anterior upper facial height
ANS-Gn	61.3 ± 3.3 mm	76 mm	72 mm	Anterior lower facial height
PNS-N	50.6 ± 2.2 mm	56 mm	51 mm	Posterior upper facial height
MP-HP	24.2 ± 5°	31°	28°	Angle of mandibular to horizontal plane
1U-NF	27.5 ± 1.7 mm	29 mm	27 mm	Distance of incisal edge of 1U to palatal plane
1L-MP	40.8 ± 1.8 mm	46 mm	46 mm	Distance of incisal edge of 1L to mandibular plane
6U-NF	23.0 ± 1.3 mm	29 mm	25 mm	Distance of mesial cusp of 6u to palatal plane
6L-MP	32.1 ± 1.9 mm	29 mm	30 mm	Distance of mesial cusp of 6l to mandibular plane

Maxilla and mandible				
ANS-PNS	52.6 ± 3.5 mm	53 mm	54 mm	Maxillary length
Ar-Go	46.8 ± 2.5 mm	58 mm	57 mm	Ramus length
Go-Pg	74.3 ± 5.8 mm	86 mm	79 mm	Mandibular length
B-Pg	7.2 ± 1.9 mm	6 mm	9 mm	Chin prominence
Ar-Go-Gn	122.0 ± 6.9°	144°	138°	Gonial angle

Dentition				
A-B	−0.4 ± 2.5°	−10.5°	0°	Distance of A to B on occlusal plane
Max1-NF	112.5 ± 5.3°	136°	121°	Angle of axis of 1U to palatal plane
Mand1-MP	95.9 ± 5.7°	86°	84°	Angle of axis of 1L to mandibular plane
